# Chest CT Scan for the Diagnosis of Pediatric Pulmonary TB: Radiological Findings and Its Diagnostic Significance

**DOI:** 10.3389/fped.2021.583197

**Published:** 2021-04-23

**Authors:** Danilo Buonsenso, Davide Pata, Emiliano Visconti, Giulia Cirillo, Francesco Rosella, Tommaso Pirronti, Piero Valentini

**Affiliations:** ^1^Department of Woman and Child Health and Public Health, Fondazione Policlinico Universitario A. Gemelli IRCCS, Rome, Italy; ^2^Dipartimento di Scienze Biotecnologiche di Base, Cliniche Intensivologiche e Perioperatorie, Università Cattolica del Sacro Cuore, Rome, Italy; ^3^Istituto di Pediatria, Università Cattolica del Sacro Cuore, Rome, Italy; ^4^Operative Unit of Neuroradiology, Surgical Department and Major Trauma, Maurizio Bufalini Hospital, Cesena, Italy; ^5^Operative Unit of Radiology, Mater Olbia Hospital, Olbia, Italy; ^6^Dipartimento Diagnostica per Immagini, Radioterapia, Oncologia ed Ematologia, Fondazione Policlinico Universitario Agostino Gemelli IRCCS, Rome, Italy; ^7^Istituto di Radiologia, Università Cattolica del Sacro Cuore, Rome, Italy

**Keywords:** tuberculosis, pediatric TB, pulmonary TB, chest computed tomography, chest X-ray, lymph node

## Abstract

Diagnosing active TB in children remains a clinical challenge, due to difficulties in achieving a definite microbiological confirmation, aspecific clinical manifestation, low sensitivity of chest radiography (CXR). For this reason, the use of chest computed tomography (CT) scan to evaluate suspected TB pediatric cases is increasing. We retrospectively reviewed records of patients aged <16 years diagnosed with active TB at the Pediatric Infectious Disease Unit of the Catholic University of the Sacred Heart to describe CT findings and to evaluate the need for its execution for diagnosis. In 41 cases, 7 CXR were normal (17.1%) while no CT scan was evaluated as negative. In 19 cases (46.3%), CXR was considered non-probable TB pulmonary, compared with 11 of 37 cases (29.7%) of CT. In 15 cases (36.6%) CXR was described as probable for TB pulmonary, instead 26 of the 37 cases evaluated by CT (70.3%) were classified as probable TB. We describe CT findings in patients with pediatric TB. We confirmed that CT can improve the diagnostic accuracy. In particular, the comparison between the CT and CXR ability in detecting cases of pulmonary TB in accordance with the proposed radiological probability criteria, showed a superiority of CT in detecting probable TB pictures (70.3%) compared with 36.6% of the x-Ray.

## Introduction

While the diagnosis of tuberculosis (TB) in adults is well-established and is based on microbiological confirmation and typical chest x-Ray findings, diagnosing active TB in children remains a clinical challenge (1–3). This population typically develop paucibacillary disease with difficulties in achieving a definite microbiological confirmation, since cultures are positive only in 25–50% of cases and sputum smear microscopy in <10–15% of children with possible TB (4–6).

Clinical manifestation may be aspecific (ranging from typical TB symptoms to just poor weight gain or astheny) and, importantly, the radiological presentation of pulmonary disease in younger children is completely different compared with the adult-type TB (5).

For these reasons, pediatric TB diagnosis relies on the experience of the clinician which need to complete a puzzle of non-specific symptoms, history of TB exposure, clinical signs, immunological tests, and radiological imaging (7, 8).

The interpretation of non-specific radiological imaging, moreover, change according to the setting the clinician is working, either high TB incidence or low-incidence, due to pre-test probability. Chest radiography (CXR) may not be sensitive in detecting lymphadenopathy, which is considered to be the fingerprint of primary pulmonary tuberculosis (7). For this reason, in high-income settings, the use of chest computed tomography (CT) scan to evaluate suspected TB pediatric cases is increasing, nevertheless no guidelines nor score are published in this regard. While a retrospective report on radiological interpretation of pediatric TB in developed countries has been recently published (9), unfortunately this study does not describe nor analyze any specific radiological findings; therefore, CXR and CT interpretation still relies only on the single radiologist experience, based on small reports currently available in literature.

For this reason, we performed this retrospective study to describe CT findings, focus on air-space involvement and lymphadenopathy.

## Materials and Methods

We retrospectively reviewed records of patients aged <16 years diagnosed with active TB at the Pediatric Infectious Disease Unit of the Catholic University of the Sacred Heart (A. Gemelli Hospital), between January 2006 and December 2015. All data were collected from medical records of patients diagnosed with active TB, and included data on demographic characteristics, clinical history, microbiology, imaging studies, medications, and outcomes. We considered foreign children those came from foreign countries. To achieve the diagnosis, we performed the following tests/procedures: tuberculin skin test (TST) performed with 5 units of Purified Protein Derivate (PPD) and induration was evaluated after 48 and 72 h. According to current international guidelines, this test was considered positive with a diameter ≥ 15 mm or less when associated with risk factors or immunodeficiency (10); quantiFERON-TB-GOLD in Tube (QTF) test; radiologic studies; examination of samples for acid-fast bacilli (AFB) was carried out by Ziehl—Neelsen staining; culture for Mycobacterium tuberculosis (MTB) on Lowenstein-Jensen medium and/or with liquid methods (BACTEC460TB or BD BACTEC MGIT 960), which were used for susceptibility testing; Polymerase Chain Reaction (PCR) (BD PROBETEC ET®, BECKTON-DICKINSON, Maryland, USA) for MTB (10–12). Gastric washing were obtained on 3 consecutive days from all patients. From those with extrapulmonary TB, samples were taken from different sites according to TB localization.

Statistical analysis was made using chi square test and *P* < 0.05 was considered statistically significant.

The research was reviewed and approved by an institutional review board (Ethics Committee of Policlinico Universitario A. Gemelli IRCCS, report number ID 2555 prot. 17791/19, 06/06/2019 Rome), and the participation involved informed consent.

### TB Definitions

Two definitions of active TB were used, as for current literature: definite (confirmed) and probable TB (8). Definite TB was defined as the presence of at least one clinical specimen positive for MTB on culture, or positive acid-fast bacilli smear microscopy, or nucleic acid amplification test positive for MTB. Probable TB was defined as the presence of three or more of the following: (1) chest radiologic findings consistent with active TB, such as lymphadenopathies associated with parenchymal airspace disease or diffuse bilateral small nodules (Miliary TB). Tuberculous pleural effusion is also characteristic, although rare in childhood; (2) typical symptoms such as fever, cough and weight loss; (3) other radiological evidence of active TB, including extra-pulmonary TB (e.g., computed tomography/magnetic resonance imaging findings consistent with TB meningitis) in conjunction with symptoms; (4) exposure to a case with active infectious TB; and (5) response to appropriate anti-TB therapy.

We then classified sites of TB as pulmonary and extrapulmonary TB.

Our inclusion criteria were: age between 0 and 16 years on the date of the diagnostic examination; final diagnosis of active confirmed or probable tubercular disease; underwent chest X-ray or CT for clinical decision.

We excluded patients with only extrapulmonary TB, immunodeficiencies, syndromic disease, incorrect execution of radiological examination.

### Imaging on Admission

While all children underwent chest X-Ray as routine part for the evaluation of a suspected TB case, chest CT scan was performed on medical decision of every single case.

Images were reviewed by three radiologists, one with 20 years and two with 5 years experience, respectively, in thoracic imaging. This review is not a normal clinical practice, but it was only performed during the study.

Chest X-rays examinations were obtained with computed radiography (CXR), in supine position. CT examinations were performed with a 64-detector-row helical CT scanner (Light Speed VCT XT, GE Medical Systems, Milwaukee, WI, USA). The following parameters were used: acquisition 64 × 0.6 mm, rotation time 0.5 s; pitch 1.20; kV 80–100; ref. mAs 45/85; reconstruction 1 mm; lter B30f/B60f. Median CTDIvol32 was 1.35. CT examination were performed with sedated patient in younger ones, in supine position, after injection of a low-osmolality, non-ionic contrast agent (370 mgI/ml) at 2 ml/kg up to 125 ml: contrast injection was mainly used to establish the mediastinal involvement of tuberculosis.

### Imaging Classification and Definitions on Diagnosis

The following CXR criteria were used to evaluate the quality of the radiographic image, both in the antero-posterior and lateral incidences: deep inhalation, correct rotation and adequate penetration of the incident beam.

We used the following parameters to define the imagines as normal, pathological probable TB (ppTB), pathological non-probable TB (pNoTB) (7).

- Normal CXR: no alteration of the normal anatomy of the thorax, possibility to visualize physiological structures not always visible (such as the thymus, visible in the pediatric age)
- ppTB CXR: a parenchymal consolidation associated with the detection of hilar lymph node or the presence of a hilar lymph node >2 cm or the detection of a miliary/disseminated micronodular pattern
- pNoTB: a CXR in which there were presumably abnormal images, but the changes were not likely to be compatible with the classical presentation of TB. For example: presence of an isolated parenchymal consolidation or a parenchymal consolidation associated with a pleural effusion; isolated pleural; detection of a micronodule or an isolated nodule; presence of an isolated lymph node <2 cm.

For the chest-CT evaluation, cross-sectional images with the parenchymal window view were used to analyze parenchymal anomalies and with the mediastinal window to identify the hilar and mediastinal adenopathies. We used the following definitions for classifications:

- normal CT: images with no alteration of the normal anatomy of the thorax
- ppTB CT: consolidation (lobar, segmental or nodular) with large lymph nodes without calcification, nor necrotic (larger than 15 mm) or cavities or military pattern or calcifying lymph nodes or necrotic lymph nodes
- pNoTB CT: pathological results not included in the above description.

## Results

### Study Population

A total of 41 patients were included in the study (Table 1): 48.8% (*n* = 20) were males and the median age was 4.68 years (range 0–16 years). In particular, 63.4% (*n* = 26) of the study population was <5 years old. Twenty nine patients (70.7%) came from foreign countries.

**Table 1:** Study population.

	**Tot**.	**X-Ray on diagnosis (%)**	**CT on diagnosis (%)**
Patients	41	41 (100)	37 (90)
Males	20	20 (48.8)	18 (48.6)
Median age	4.683		
Nationality Italians	12	12 (29.3)	11 (29.7)
Symptomatic TB	35	35 (85.4)	31 (83.8)
Confirmed TB	34	34 (82.9)	30 (81.1)
Probable TB	7	7 (17.1)	7 (18.9)
Contact +	11	11 (26.8)	10 (27)
Smear +	13	13 (31.7)	13 (35.1)
Culture +	25	25 (61)	23 (62.2)
PCR +	25	25 (61)	22 (59.4)
Fever	28	28 (68.3)	24 (64.9)
Weight loss	10	10 (24.4)	9 (24.3)
Night sweating	3	3 (7.3)	2 (5.4)
Cough	22	22 (53.6)	21 (56.7)
Drug resistance (1 or more)	3	3 (7.3)	3 (8.1)

85.4% of the children (*n* = 35) were symptomatic. Among the systemic symptoms, the most frequent were fever, identified in 28 children (68.3% of cases), and weight loss, reported in 10 children (24.4%). Other systemic symptoms were nocturnal sweating (7.3%) and asthenia (4.8%).

Among the respiratory symptoms, the most frequent was cough, reported in 22 children (53.6%). Other symptoms included respiratory failure (2.4%) and dyspnea (2.4%).

The TST was performed in 35 cases and it was positive in 32 children, negative in 3 and not reported or not carried out in 6 cases. The QTF test was performed in 13 children and it was positive in 11 patients. In one case the test was negative and in another child it resulted indeterminate.

Pulmonary TB was confirmed in 34 cases (82.9%) while it was probable in 7 children (17%). The microbiological diagnosis was made by PCR of respiratory samples or on the gastric aspirate and was positive in 25 children (61% of cases). The cultures were also positive in 25 children, while the bacteriological smear was positive for alchool-acid resistent bacteria in 13 cases (31.7%). Resistance to antibiotic therapy was found in 3 patients (7.3%).

### CT Findings

Table 2 shows details about the CT findings. In 4 patients chest CT was not performed because the parents did not give their consent. In 27 cases (73%) consolidation areas were found, with a greater frequency in the group of children under 5 years of age. Four types of consolidation patterns have been described. The segmental pattern was present in 40.5% (15) of cases; segmental with center-lobar nodule (*n* = 8, 21.6%), which was more frequent in children over 5 years of age (6 cases) (*p* < 0.01); nodular and lobar consolidation patterns [each noted 6 cases (16.2%)].

**Table 2:** CT findings.

**CT scan**	**Tot. (37)**	** <5 years (23)**	**≥ 5 years (14)**	***P***
Consolidations (tot.)	27 (73.0)	18 (78.3)	9 (64.3)	Ns
- Segmental	15 (40.5)	13 (56.5)	2 (14.3)	0.04
- Nodular	6 (16.2)	5 (21.7)	1 (7.1)	Ns
- Lobar	6 (16.2)	3 (13.0)	3 (21.4)	Ns
- Segmental + nodular centrolobular	8 (21.6)	2 (8.7)	6 (42.8)	<0.01
Nodules	13 (35.1)	4 (17.4)	9 (64.3)	<0.01
Centrolobular nodules	8 (21.6)	2 (8.6)	6 (42.8)	0.03
Calcific nodules	2 (5.4)	1 (4.3)	1 (7.1)	Ns
Tree-in-bud	5 (13.5)	0 (0)	5 (35.7)	<0.01
Cavernae	7 (18.9)	3 (13.0)	4 (28.6)	Ns
Bronchial thickening	11 (29.7)	5 (21.7)	6 (42.8)	Ns
Miliary pattern	4 (10.8)	3 (13.0)	1 (7.1)	Ns
Pleural thickening	6 (16.2)	4 (17.4)	2 (14.3)	Ns
Lymphadenopathy	33 (89.2)	21 (91.3)	12 (85.7)	Ns
- Prevasculars	5 (13.5)	3 (13.0)	2 (14.3)	Ns
- Hilar	30 (81.1)	20 (86.9)	10 (71.4)	Ns
- Paratracheal (inf)	23 (62.2)	13 (56.5)	10 (71.4)	Ns
- Paraortic	10 (27.0)	4 (17.4)	6 (42.8)	Ns
- Subcarenal	11 (29.7)	6 (26.1)	5 (35.7)	Ns

*NS, Not significant*.

Among the other parenchymal anomalies, the nodules were reported in 13 cases (35.1%), 9 in the group with more than 5 years (64.3%) (*p* < 0.01). Bronchial thickening was noted in 11 cases (29.7%). Cavitations were less frequently found (*n* = 7, 18.9%).

In 5 cases the nodular tree-in-bud pattern was found all in children > 5 years of age (*p* < 0.01); the miliary pattern was present in 4 children (10.8%, three of them were children under 5 years of age). Pleural involvement was found in 6 cases (16.2%).

A lymphadenopathy was found in 33 children (out of 37 children who underwent CT scan), with generally multiple locations (see Table 2 for details). It was found as an isolated lesion, without other associated parenchymal anomalies, in 4 cases (10.8%), all in children under the age of 5 years.

Nine children (24.3%) had lymphadenopathy <10 mm in size, 18 children had at least one lymphadenopathy between 10 and 20 mm (48.6%), in 6 cases (16.2%) at least one lymphadenopathy was 20 mm or more. Among the microbiologically confirmed cases, 7 (23.3%) had a size of <10 mm, 16 (53.3%) between 10 and 20 mm, 4 (13.3%) dimensions > 20 mm, 3 cases (10%) had no lymphadenopathy. Details about lymph node size according to age are described in Table 3.

**Table 3:** Lymph-nodal characteristics after contrast administration.

**Characteristics of adenopathies**	**Tot (37)**	** <5 (23)**	**≥ 5 (14)**	**Confirmed TB (30)**	**Probable TB (7)**	***P***
Ring	6 (16.2)	4 (17.3)	2 (14.3)	4 (13.3)	2 (28.6)	Ns
Non-homogeneous	1 (2.7)	-	1 (7.1)	1 (3.3)	-	Ns
Homogeneous	15 (40.5)	10 (43.5)	5 (35.7)	11 (36.7)	4 (57.1)	Ns
Calcifications	10 (27)	5 (21.7)	5 (35.7)	7 (23.3)	3 (42.9)	Ns
Pointed	4 (10.8)	2 (8.7)	2 (14.3)	3 (10)	1 (14.3)	Ns
Shell	5 (13.5)	3 (13.4)	2 (14.3)	3 (10)	2 (28.6)	Ns
Moriform	1 (2.7)	-	1 (7.1)	1 (3.3)	-	Ns
Size						Ns
<1 cm	9 (24.3)	6 (26.1)	3 (21.4)	7 (23.3)	2 (28.6)	Ns
1-2	18 (48.6)	12 (52.2)	6 (42.8)	16 (53.3)	2 (28.6)	
>2 cm	6 (16.2)	3 (13.0)	3 (21.4)	4 (13.3)	2 (28.6)	
NO linf	4 (10.8)	2 (8.7)	2 (14.3)	3 (10)	1 (14.3)	
Lymphnode as only pathological finding	4 (10.8)	4 (17.3)	-	2 (6.67)	2 (28.6)	Ns

*NS, Not significant*.

We also evaluated lymph nodal enhancement after contrast administration according to age (details reported in Table 3). Four children younger than 5 years presented a classic ring enhancement pattern (17.3%) against 2 children over 5 years of age (14.3%); 10 children aged <5 years had a homogeneous enhancement (43.5%) vs. 5 (35.7%) of a higher age. Calcifications were present in 5 children younger than 5 years and (21.7%) and 5 older than 5 years (35.7%). In particular, point calcifications were found in 2 cases of children younger than 5 years (8.7%) and 2 (14.3%) over the age of 5.

## Discussion

We have analyzed radiological patterns in 41 children with pulmonary TB, describing in details air-way space and lymph nodal characteristic, and analyzing age-related and microbiological differences. Microbiological confirmation of tuberculosis is difficult in the early stages, and the presence of recent contacts, immunological findings, and suggestive radiological signs allow diagnosis in most cases.

About radiology, chest X-ray is the first level examination and it is characterized by the presence of lymphadenopathy with or without parenchymal involvement. However, this exam has poor sensitivity in the study of lymphadenopathy, a hallmark of tuberculosis in children.

A new technique is represented by lung ultrasound (LUS): a score system that combines X-ray and LUS will probably be validated in the near future. Some pediatric studies clarified the accuracy of LUS in bacterial pneumonia (13), while its role in pediatric TB is still under evaluation (14, 15).

MRI is a radiation-free alternative to CT. However, its use is limited due to the high cost and sedation required, especially for those regions where TB is endemic (16).

Our study raises an important question: the role of CT in the diagnosis of pediatric TB. Although it is a more sensitive test for detecting lymphadenopathy, it uses ionizing radiation and may require the use of contrast and sedation in the younger patient.

Since low-dose techniques comparable to the dose of a few chest radiographs and the current absence of an alternative method have developed in recent years, this test should be used in groups where first-level examinations are inconclusive and clinical suspicion is high.

### The Diagnostic Significance of CT Scan

The comparison between the CT and CXR ability in detecting cases of pulmonary TB in accordance with the proposed radiological probability criteria, showed a superiority of CT in detecting probable TB pictures (70.3%) compared with 36.6% of the x-Ray. Also, we found 7 (17.1%) normal CXRs showing CT anomalies; in both cases the difference was statistically significant (*p* < 0.01); moreover, the presence of pulmonary adenopathies was described in 89.2% of CT scan (33/37) while only in 36.6% (15/41) at CXR. Our data are in line with those reported in the literature (17–23).

### Lymph-Nodal Involvement

In children, especially in the younger ones, the physiological thymic protrusion limits the potential of plain radiographs. The enlarged lymph nodes become evident only when projecting over the heart and the thymus. Lateral projection improves accuracy, but this projection is not always easy to perform in younger children and infants. In this situation, particularly in order to study lymph-nodal disease, CT is useful.

In our study, the most frequently anomalies were adenopathies (89.2%), first among those hilar (81.1%) followed by the lower paratracheal (62.2%) with usually multiple localizations: in only two cases we found a single localization. Adenopathies were the most frequent pathological finding also in the study by Kim et al. but they found a more frequent right paratracheal localization compared to the hilar (20). Still different distributions were described by Mukund et al. with 84% of paratracheal adenopathies followed by sub-carval localization (76.1%), while the right hilar was described only in 52.3% of the cases (22).

Tomà et al. recently published a series of 113 pediatric cases of lung TB, 69 were under 5 years of age and 44 over 5 years of age (9). All the cases aged under 5 years had a lymph node disease. Out of the 44 cases aged over 5 years, only four patients had lymphadenopathies. Unfortunately, they did not give a strict description of definition, size, localization, morphology and statistical analyses. This differs from our series, where two children in the <5 years old group had no lymph-nodal disease also on CT scan. This is an important finding since lymph-nodal disease, despite a recognized sign of thoracic TB in children, is not 100% sensitive also in younger children.

Regarding the size of adenopathies, in our study we found that 24 patients (64.8%) had lymphadenopathy with a diameter ≥10 mm, while 9 (24.3%) had lymph nodes smaller or equal to 10 mm; 4 (10.8% of total patients) had no visible lymphadenopathy.

The typical lymph nodal enhancement pattern after contrast administration was the peripheral ring pattern, but two additional patterns were also described in the literature: homogeneous and inhomogeneous (17, 22, 24). In our study, the most common pattern was the homogeneous one, present in 15 cases (40.5%), followed by the peripheral ring enhancement with central hypodensity in 16.2% (6/37) while one single case showed inhomogeneous pattern. The frequencies found in the other studies were completely discordant. In Kim et al. 70.7% of patients had lymphadenopathy with ring enhancement, while in Mukund et al. the most commonly found pattern was the inhomogeneous one with 52.3% of cases, followed by the homogeneous one (34.1%) and only 13.6% of cases had peripheral ring enhancement (20, 22). Calcifications were present in 10 children out of the total of 37 (27%) assessed by CT in our study. This finding was in line with other studies (20). In the largest series published so far, enhancement patterns were unfortunately not described, so our series remains an important reference point for clinicians (9).

Children older or younger than 5 years of age showed no differences in terms of localization, morphology and size of adenopathies.

### Air-Space Involvement

Consolidations represented the most frequently described parenchimal abnormality.

According to Tomà et al. consolidations had a segmental/lobar distribution with, unlike adults, a lower and middle lobe predominance, similarly to our series (9).

Moreover, the parenchymal lesions type tree-in-bud were more frequent in children older than 5 years. These findings were similar to those described by Kim et al. (20).

In our study, 7 children (18.9% of cases evaluated by CT) showed cavitations, in line with current literature, confirming that this radiological pattern is rare in pediatric patients. We know that cavitations, a sign of high infectivity and a high bacterial load, are the pathognomonic radiological finding of adult-type TB and therefore tend to be rare in children, while affect about 45% of the adult TB patients.

The last pattern to be evaluated for parenchymal lesions was the miliary, found in our study in 4 children (10.8% of cases). Three of these cases (13%) were described in children under the age of 5, while only one in the age group over 5. It should be noted, however, that this child was 5 years and a few months at the time of the diagnosis. Although the difference between the two groups was not statistically significant, it is possible to think of a greater tendency for younger children to develop pictures of lung pulmonary TB.

Pleural involvement occurs after direct spread of caseous material from a sub-pleural parenchymal or lymph node focus, or from haematogenous spread. Usually, it represents a hypersensitivity response (18). Serous involvement is not frequent in pediatric patients. Tomà et al. described 8 cases out of 146 under 5 years of age, 5 cases out of 76 under 2 years, and 18 cases out of 71 over 5 years (9). In our study, a pleural involvement was described in 9 cases (24.3%), 3 cases of pleural effusion and 6 of pleural thickening. This distinction is possible only through TC evaluation, allowing the detection of pleural effusion, thickening of the visceral and parietal pleura (“split-pleura sign”), pleural nodularity, and pleural rind (25). On CXR, the number of cases detected in our study was lower (6 cases, 14.6%).

Our study has several limitations. The retrospective nature is an inherent limitation that may have led to the lack of information. Second, use of chest CT was based on the physician's decisions and not on standardized local protocols, not allowing us to adequately define the role of the exam in our children. However, our study provided a broad, detailed, and comprehensive description of the involvement of airspace and lymphadenopathy in pulmonary TB.

## Conclusion

Pulmonary TB in pediatric age differs from adult TB in the presence of lymph node and parenchymal disease. However, CXR has poor sensitivity in the study of lymphadenopathy.

Chest radiography continues to be used for the initial evaluation of children with suspected TB, but CT can improve the diagnostic accuracy.

Unfortunately, we were unable to provide a statistically significant score for chest CT finding in diagnosing pediatric active TB due to the limited number of patients. This scoring system will require further evaluation in larger studies.

## Data Availability Statement

The original contributions presented in the study are included in the article/supplementary material, further inquiries can be directed to the corresponding authors.

## Ethics Statement

The studies involving human participants were reviewed and approved by Ethics Committee of Policlinico Universitario A. Gemelli IRCCS, report number ID 2555 prot. 17791/19, 06/06/2019 Rome. Written informed consent to participate in this study was provided by the participants' legal guardian/next of kin.

## Author Contributions

DB, PV, and TP contributed conception and design of the paper. DB and DP wrote the manuscript. GC collected the data. TP, EV, and FR analyzed CT images. All authors contributed to manuscript revision, read, and approved the submitted version.

## Conflict of Interest

The authors declare that the research was conducted in the absence of any commercial or financial relationships that could be construed as a potential conflict of interest.
